# Bacterial infections exacerbate myeloma bone disease

**DOI:** 10.1186/s12967-021-03187-5

**Published:** 2022-01-06

**Authors:** Rui Liu, Yuping Zhong, Rui Chen, Shiyi Chen, Yazhu Huang, Huan Liu

**Affiliations:** 1 Cancer Research Center, School of Medicine, Xiamen University, Xiamen, 361102 China; 2 Department of Hematology, Qingdao Municipal Hospital, School of Medicine, Qingdao University, Qingdao, 266011 China

**Keywords:** Multiple myeloma, Bone lesion, *Escherichia coli*, Lipopolysaccharides

## Abstract

Multiple myeloma is characterized by osteolytic lesions caused by reduced bone formation and activated bone resorption. An important feature of myeloma is a failure of bone healing after successful treatment. In this work, clinical studies indicated a highly positive correlation between bone marrow bacteria abundance and bone lesion numbers of myeloma patients in complete remission. Coculture experiments demonstrated that marrow *Escherichia coli* (*E. coli*) promotes osteoclast differentiation and inhibits osteoblast differentiation. Mechanism studies showed that *E. coli* lipopolysaccharides (LPS) activated NF-κB p65 signaling and reduced phosphorylated smad1/5/9 binding ability with *RUNX2* promoter, leading to decreased RUNX2 expression in osteoblast progenitors. Additionally, LPS enhanced phosphorylated NF-κB p65 binding ability with *NFATc1* promoter, leading to increased NFATc1 expression in osteoclast progenitors. In vivo studies revealed *E. coli* contributes to osteolytic bone lesion, and elimination of *E. coli* infection assists healing of bone lesion in mouse model of myeloma in complete remission. These findings establish a heretofore unrecognized effect for *E. coli* in the genesis of myeloma bone disease and suggest a new treatment strategy.

**Supplementary Information:**

The online version contains supplementary material available at 10.1186/s12967-021-03187-5.

## Introduction

Multiple myeloma (MM) is a B cell malignancy within bone marrow and remains an incurable disease. Majority of patients with myeloma develop bone destruction that causes severe bone pain, pathological fractures, hypercalcemia and spinal cord compression [1]. Bone remodeling is a process where osteoblasts form bone and osteoclasts resorb bone [2]. This delicate balance is interrupted in some types of cancers, including breast cancer, lung cancer and myeloma [3, 4]. In myeloma, bone disease remains incurable, even in complete remission patients, there is no plasma cells in their bone marrow microenvironment, but bone lesions still do not heal. The mechanisms underlying are still poorly understood [5]. In active myeloma patients, factors secreted by myeloma cells can disrupt the balance between osteoclast-regulated bone resorption and osteoblast-regulated bone formation. For example, myeloma cells stimulate production of cytokines such as macrophage inflammatory protein-1α (MIP-1α), RANKL and monocyte chemoattractant protein-1 (MCP-1) and thus enhance osteoclast bone resorption activity [3, 6]. On the other hand, myeloma cells secrete dickkopf-related protein 1 (DKK1) inhibits the Wnt/β-catenin signaling pathway and suppresses differentiation of mesenchymal stem cells (MSCs) into osteoblasts [7]. When patients are in remission, lytic lesion is expected to heal. Unfortunately, bone lesion still exists in the patients, which indicates that some other factors within the bone marrow microenvironment leads to the failure of bone healing.

To identify more potential factors involved in the maintenance of myeloma patient bone lesion in complete remission, we focused on the interactions between tumor microenvironment and bacteria, which has become a novel field of research in recent years. It has been evident that bacteria modulate tumor immunotherapy, drug resistance and tumorigenesis [8–13]. In metastatic melanoma, the commensal bacteria are associated with anti-PD-1 efficacy [12]. In colon cancer, intratumor bacteria mediate tumor resistance to the chemotherapeutic drug gemcitabine [11], and *Fusobacterium nucleatum* promotes chemotherapy drug resistance by regulating autophagy [13]. In leukemia, bacterial signals are critical for the development of pre-leukemic myeloproliferation in tet methylcytosine dioxygenase 2 deficient expression hematopoietic cells [10]. Additionally, in myeloma, bacterial infections represent a major threat to myeloma patients and cause early death among patients [14]. However, it is still unknown whether bacterial infection has any role in myeloma-induced osteolytic bone disease and whether antibiotics could render bone lesions curable.

In this study, we observed that bone marrow *E. coli* promotes the persistence of myeloma-associated bone lesions via LPS that activates bone resorption and suppresses bone formation. Our findings provide a potential treatment approach for complete remission patients with bone lesions by targeting bone marrow bacterial infections.

## Results

### Association of bone marrow bacteria with bone lesion in patients of myeloma in remission

**Fig. 1:**
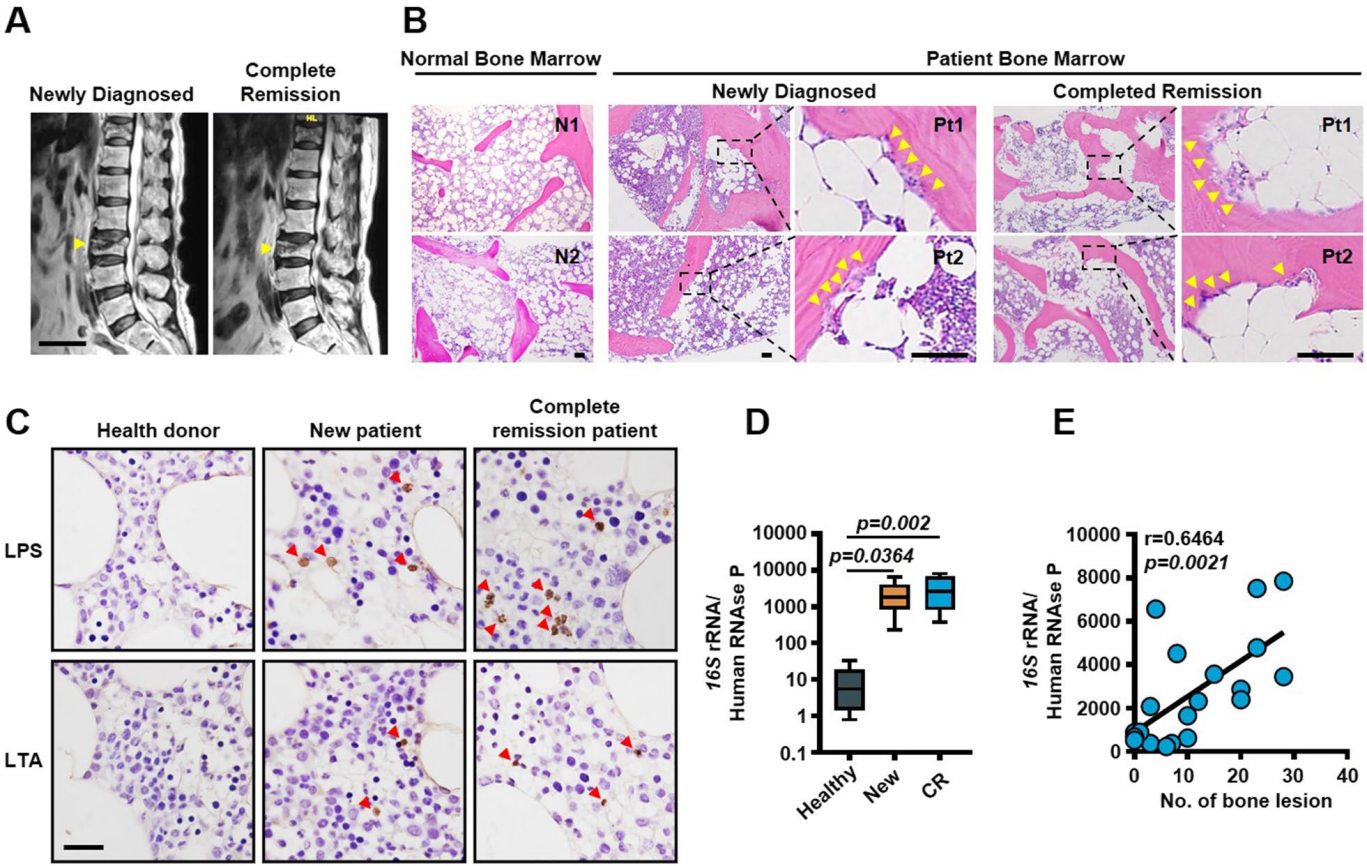
Association of bone marrow bacteria with bone lesion in patients with myeloma. **A** Magnetic resonance imaging scanning for myeloma patient lytic lesions in the spine. Arrows, lytic lesions. Scale bars, 5 cm. **B** HE staining sections of normal bone marrow (BM) or the images and close-up views of local destruction in the BM from two myeloma patients at the time of diagnose and at remission. Arrows, lytic lesions. Scale bars, 100 μm. **C** Immunohistochemical examination for LPS (Gram-negative bacteria marker) or LTA (Gram-positive bacteria marker) levels of normal bone marrow or the bone marrow from the patient with myeloma. Arrows, Bacteria. Scale bars, 50 μm. **D** qPCR analysis of healthy, newly diagnosed (New) and complete remission (CR) myeloma patients bone marrow aspirates bacterial *16S* rRNA levels, as compared to DNA copies of host human RNase P in order to calculate the relative bacterial amount per biopsy sample relative to amount of host tissue. All *P* values were determined using one way ANOVA. **E** Correlation coefficient of *16S* rRNA levels and the numbers of bone lesion in myeloma patients (n = 20). The correlations were evaluated using Pearson coefficient. r, correlation coefficient. *P* value was determined by Pearson coefficient

We found an intractable problem in the treatment of myeloma: osteolytic bone lesions do not heal, even the patient is in complete remission (Fig. 1A, B). Immunohistochemistry examination of bone marrow biopsy samples obtained from myeloma patients (active and remission stage) showed presence of gram-negative (LPS^+^) and gram-positive (LTA^+^, Lipoteichoic acid) bacteria compared to healthy bone marrow (Fig. 1C). To determine the abundance of bacteria in bone marrow under physiological conditions, the bacterial ribosomal *16S* rRNA gene was amplified by a real-time quantitative PCR (qPCR) assay with universal primers. We detected much more bacteria in the bone marrow of myeloma patient in active and remission, but less in healthy donors (Fig. 1D). We also found a robust correlation between bone marrow bacteria abundance and bone lesion numbers (Fig. 1E). Therefore, bone marrow bacteria is likely to be a key regulator of bone lesion in myeloma patients.

### *Escherichia coli* inhibits osteoblastogenesis and promotes osteoclastogenesis

Bone remodeling is regulated by a balance between osteoclast-mediated resorption and osteoblast-mediated matrix synthesis. To examine whether bone marrow bacteria can regulate this balance, we first assessed their effects on osteoblast and osteoclast differentiation.

**Fig. 2:**
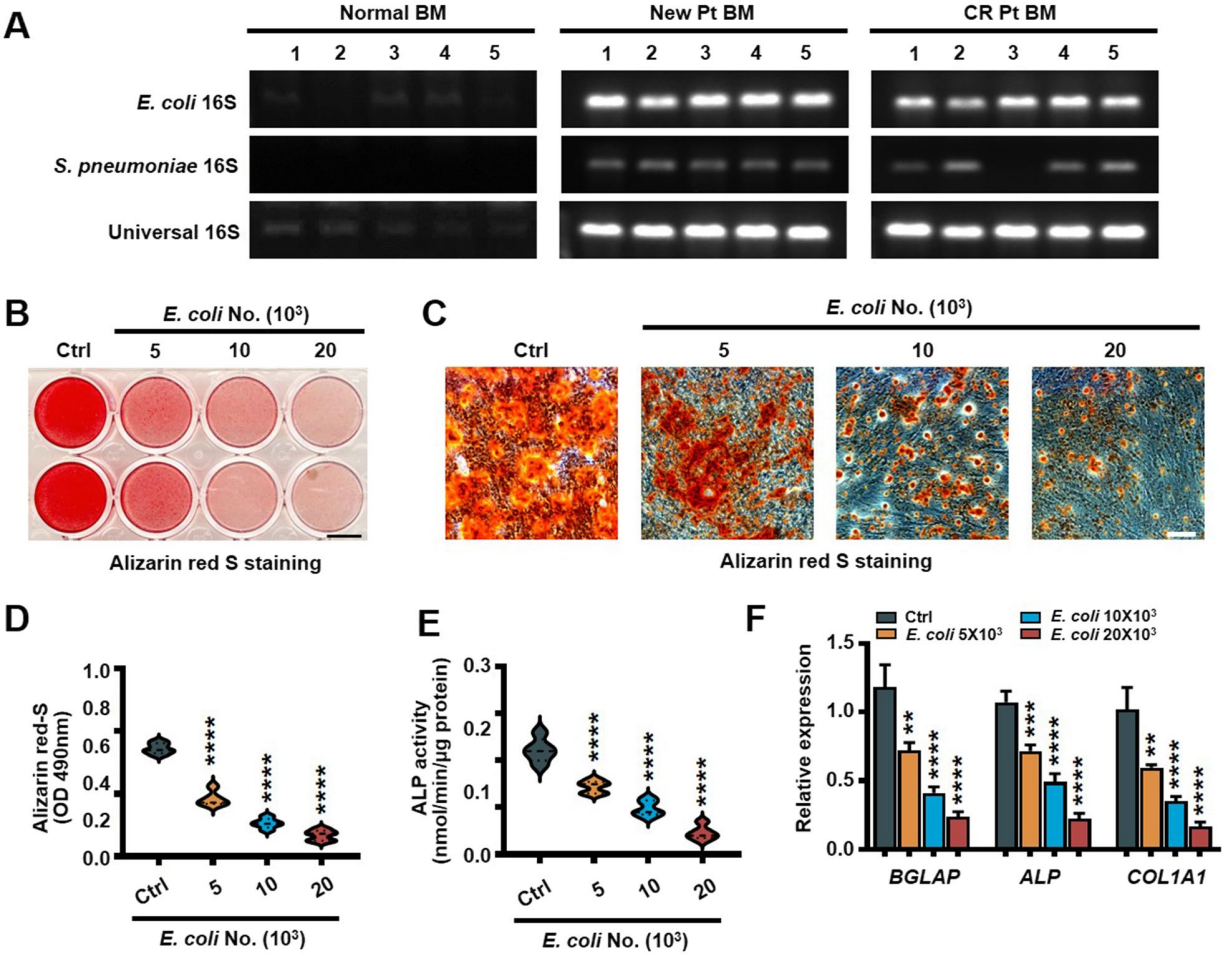
*Escherichia coli* inhibits osteoblastogenesis. **A** The expression of universal, *S. pneumoniae* and *E. coli 16S* rRNA in healthy, newly diagnosed and complete remission myeloma patients bone marrow aspirates (n = 5). MSCs were cocultured with *E. coli* in osteoclast medium. Shown are representative images (**B**, **C**) and summarized results (**D**) of Alizarin red S staining, ALP activity (**E**), and the relative expression of osteoblast marker genes (*BGLAP*, *ALP* and *COL1A1*) genes (**F**). Scale bars, 7 mm (**B**) and 100 μm (**C**). Data are averages ± SD. Each experiment was repeated three times. **P < 0.01, ***P < 0.001; ****P < 0.0001. All *P* values were determined using one way ANOVA

**Fig. 3:**
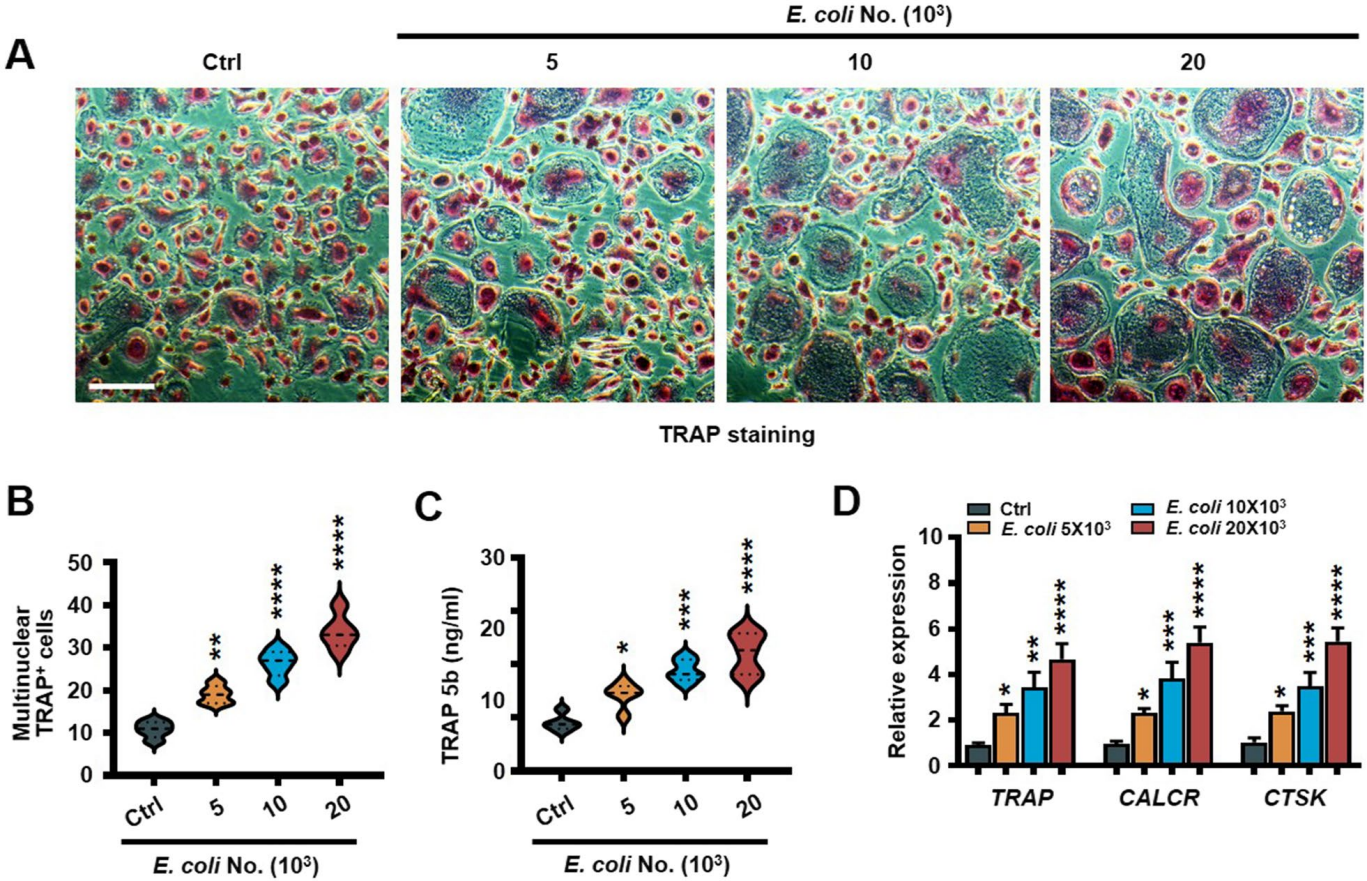
*Escherichia coli* promotes osteoclastogenesis. Precursors of osteoclasts were cocultured with *E. coli* in osteoclast medium. Shown are the morphologies (**A**) and the numbers of multinuclear (≥ 3) TRAP^+^ mature osteoclast cells (**B**), the amount of secreted TRAP 5b (**C**) and relative expression of the osteoclast marker genes (*TRAP*, *CALCR*, and *CTSK*) (**D**). Scale bars, 100 μm. Data are averages ± SD. Each experiment was repeated three times. *P < 0.05; **P < 0.01, ***P < 0.001; ****P < 0.0001. All *P* values were determined using one way ANOVA

Previous study identified that *E. coli* and *Streptococcus pneumoniae* (*S. pneumoniae*) are the most frequent pathogen in myeloma patients bone marrow [15]. We observed more *E. coli*-but not *S. pneumoniae* in bone marrow aspirates of myeloma patient in active and remission (Fig. 2A). In osteoblast differentiation assay, we cocultured MSCs (osteoblast progenitors) in osteoblast medium with *E. coli*. MSCs cultured alone in this medium served as a positive control. We observed significantly lower Alizarin red S staining (Fig. 2B–D), alkaline phosphatase activity (Fig. 2E) and osteoblast marker genes expression [bone gamma-carboxyglutamic acid-containing protein (*BGLAP*), alkaline phosphatase (*ALP*), and collagen type I α1 (*COL1A1*)] (Fig. 2F) in MSCs cultured with *E. coli*. In osteoclast differentiation assay, we cocultured precursors of osteoclasts (preOCs) with *E. coli*, which stimulated RANKL-induced TRAP^+^ cell formation (Fig. 3A, B), TRAP 5b secretion in the supernatant (Fig. 3C) and osteoclast marker genes expression [tartrate-resistant acid phosphatase (*TRAP*), cathepsin K (*CTSK*) and calcitonin receptor (*CALCR*)] (Fig. 3D). Our aggregated results demonstrated that *E. coli* enhances osteoclastogenesis and suppresses osteoblastogenesis.

### *Escherichia coli* LPS inhibits osteoblastogenesis and enhances osteoclastogenesis through NF-κB signaling

**Fig. 4:**
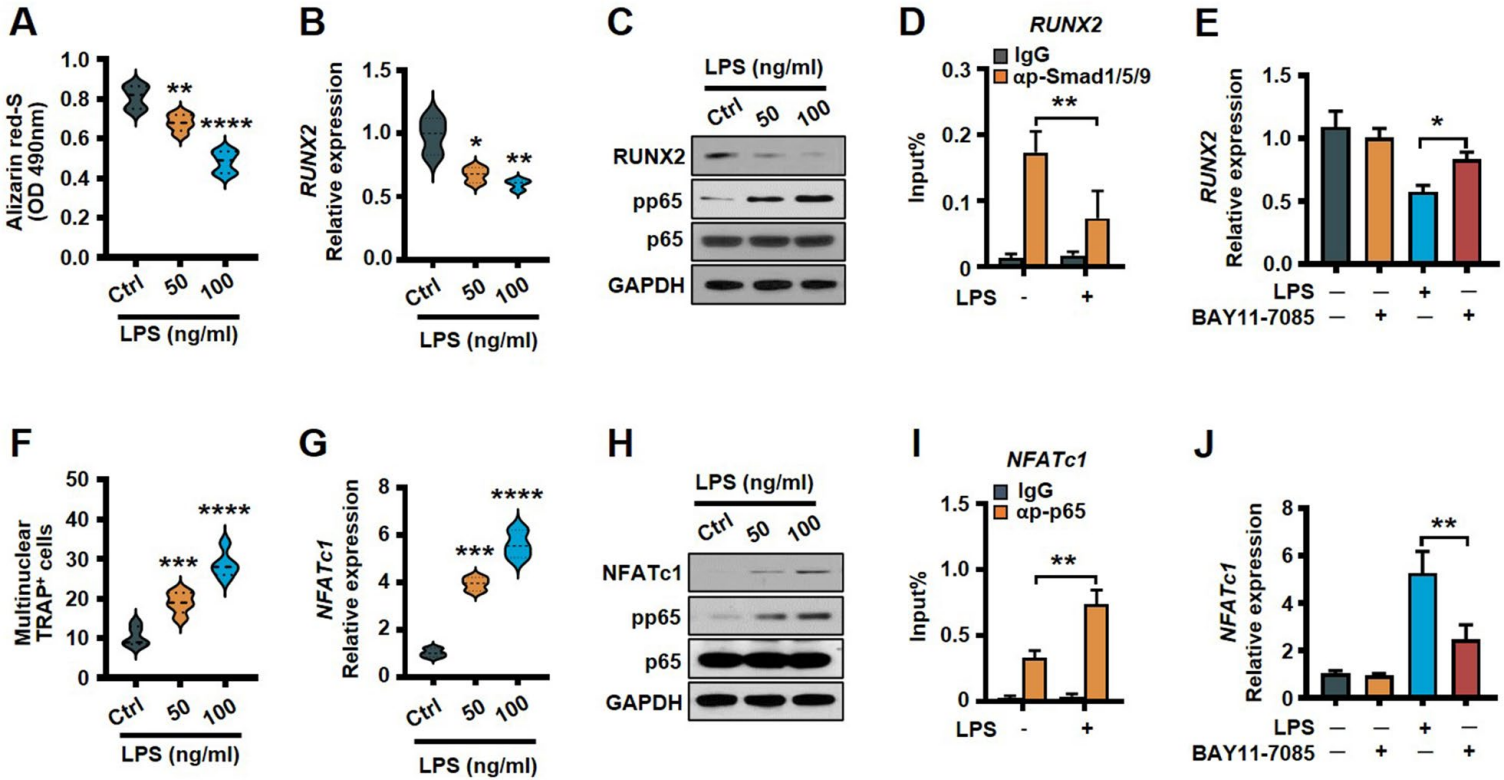
*Escherichia coli* lipopolysaccharides inhibits osteoblastogenesis and enhances osteoclastogenesis through NF-κB signaling**.** MSCs were treated with or without LPS, after culture, the cells were assayed for Alizarin red S staining (**A**). qPCR analysis for *RUNX2* mRNA levels (**B**) and western blot analysis showing the expression of RUNX2, non-phosphorylated and phosphorylated (p) p65 (**C**). **D** ChIP assay for the interaction of p-smad1/5/8 with promoter of *RUNX2* using anti-p-smad1/5/8 antibody or rabbit IgG. The input proteins served as controls. **E** Expression of *RUNX2* in MSCs treated with or without LPS (100 ng/ml) or BAY11-7085 (NF-κB inhibitor) (10 μM). PreOCs were treated with or without LPS, after culture, the cells were assayed for TRAP staining (**F**), qPCR analysis of *NFATc1* mRNA levels (**G**) and western blots showing the expression of NFATc1, non-phosphorylated and phosphorylated p65 (**H**). **I** ChIP assay for the interaction of p-p65 with promoter of *NFATc1* using anti-p-p65 antibody or rabbit IgG. The input proteins served as controls. **J** Expression of *NFATc1* in preOCs treated with or without LPS (100 ng/ml) or BAY11-7085 (10 μM). Data are averages ± SD. Each experiment was repeated three times. *P < 0.05; **P < 0.01, ***P < 0.001; ****P < 0.0001. All *P* values were determined using one way ANOVA

We next examined the signaling pathways by which *E. coli* may regulate bone destructions. Previous work has established that bacteria implicated in bone diseases contain or produce molecules with potent effects on bone cells. Bacterial factors can interact with osteoblast or the osteoclast to promote bone resorption and inhibit bone formation [16]. LPS is an important pathogenic factor from gram-negative bacteria cell wall, and LPS-TLR4 (Toll like receptor 4) complex has been identified as one of regulators involved in bone remodeling [17, 18], but the downstream signaling are not very clear. Consistent with previous study, osteoblastogenesis assay indicated that LPS inhibits osteoblast differentiation (Fig. 4A). Runt related transcription factor 2 (RUNX2) is a key regulator of osteoblast differentiation. qPCR and western blot analysis showed that MSCs *RUNX2* mRNA and protein levels are lower treated with LPS as compared to untreated cells (Fig. 4B, C). Bone morphogenetic proteins (BMPs) 2 are members of the transforming growth factor β superfamily (TGF-β) that were originally demonstrated to promote bone formation. BMPs activate downstream smad1/5/8 signaling, and the three smads form heteromeric complexes with smad4, which translocate into the nucleus and activate the transcription of target genes, including RUNX2 [19]. To further explore the potential LPS downstream signaling pathways, we treated MSCs with different concentrations of LPS and found that LPS enhanced phosphorylation of NF-κB (nuclear factor κB) p65 signaling (Fig. 4C). Chromatin Immunoprecipitation (ChIP) assay using an anti-p-smad1/5/9 antibody showed LPS-activated NF-κB reduced p-smad1/5/9 binding ability with *RUNX2* promoter (Fig. 4D). To show the specificity of NF-κB signaling pathways on RUNX2 inhibition, we treated *E. coli* and MSCs coculture system with BAY11-7085, a NF-κB signaling inhibitor, resulting in up-regulation of RUNX2 expression (Fig. 4E).

In osteoclastogenesis assay, LPS promotes osteoclast differentiation (Fig. 4F). qPCR and western blot analysis showed that LPS increased preOCs *NFATc1* mRNA and protein levels as compared to untreated cells (Fig. 4G, H). Nuclear factor of activated T-cells, cytoplasmic 1 protein (NFATc1) is essential for osteoclast differentiation [2]. Western blot analysis showed that LPS enhanced phosphorylation of NF-κB p65 (Fig. 4H). ChIP assay using an anti-p-p65 antibody showed LPS enhanced phosphorylated NF-κB p65 binding ability with *NFATc1* promoter (Fig. 4I) and promotes its transcription. Furthermore, NF-κB signaling inhibitor BAY11-7085 reduced LPS-induced NFATc1 expression (Fig. 4J). Together, these findings indicated that *E. coli* LPS inhibits osteoblastogenesis and enhances osteoclastogenesis through NF-κB p65 signaling.

### *Escherichia coli *contributes to osteolytic bone lesion and elimination of *E. coli* heals resorbed bone in mouse model of myeloma in remission

**Fig. 5:**
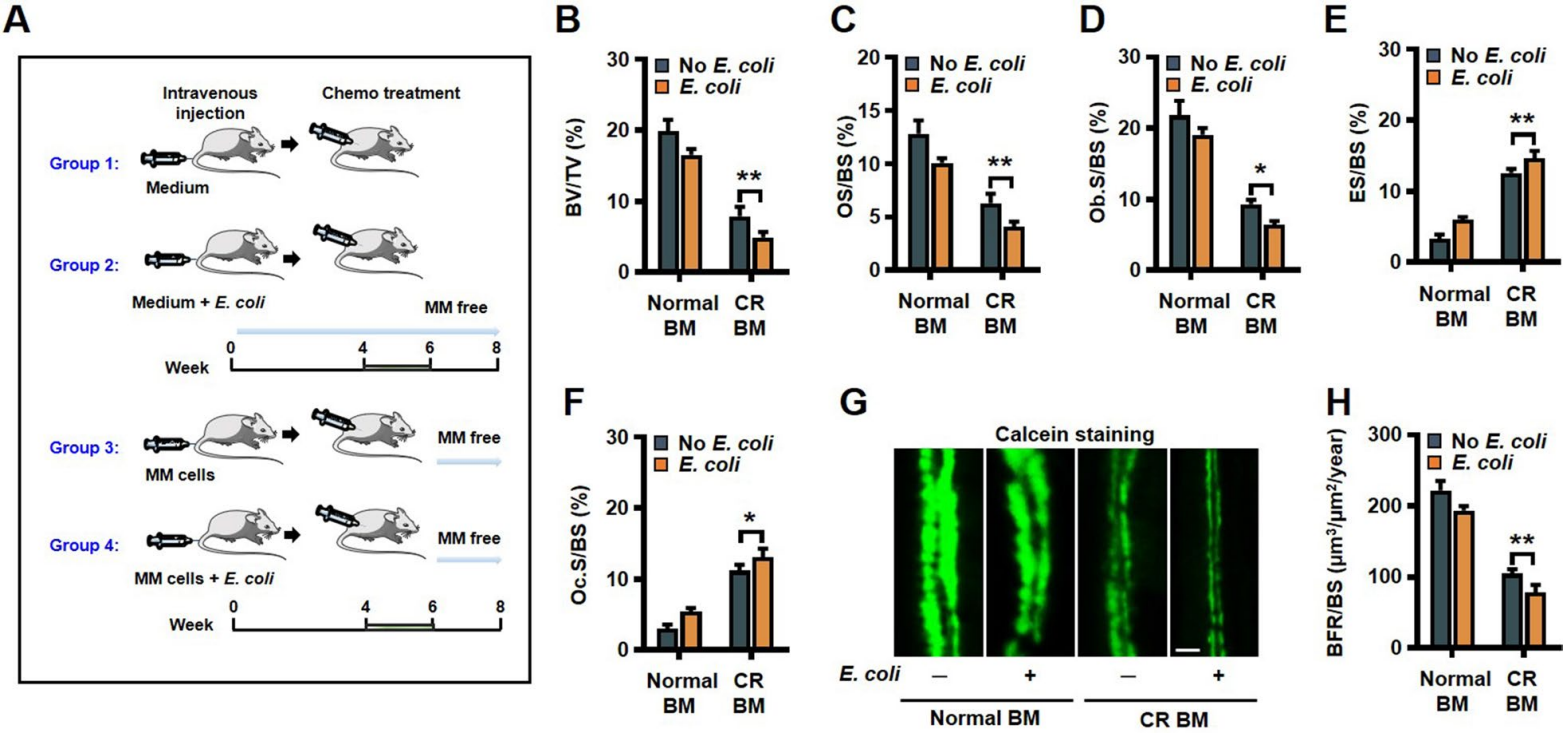
*Escherichia coli* contributes to osteolytic bone lesion in a mouse model of myeloma in complete remission. **A** C57BL/KaLwRij mice were intravenous injected with the 5TGM1 (5 × 10^5^ cells/mouse) with or without *E. coli* (1 × 10^4^ cells/mouse). After 4 weeks, 1 mg/kg bortezomib and 2 mg/kg melphalan were injected intraperitoneally into the mice (3 times a week) for 2 weeks. Histomorphometric analysis of BV/TV (**B**), OS/BS (**C**), Ob.S/BS (**D**), ES/BS (**E**) and Oc.S/BS (**F**). **G**, **H** Bone formation rate (BFR/BS) was measured by calcein injection, and the bone sections were imaged and analyzed. Shown are representative images and summarized data of bone formation in mouse femurs. Scale bar: 20 μm. Data are means ± SD (*n* = 5 mice/group, three replicate studies). *P < 0.05; **P < 0.01. *P* values were determined using one-way ANOVA

According to our previous study [20], we constructed a myeloma mouse model in complete remission with or without *E. coli* infection. We intravenous injected myeloma cells 5TGM1 with or without *E. coli*, treated the mice with chemotherapy; mice not injected with myeloma cells but injected with or without *E. coli* served as controls (Fig. 5A). Two weeks after chemotherapy drugs treatment, M-protein, an indicator of myeloma burden, fell to a very low level, which is consist with our previous results. This suggests that the chemotherapy drugs-treated mice were almost or totally myeloma-free. After 8 weeks, in complete remission myeloma mice with *E. coli* infection, bone histomorphometric analysis indicated a lower percentages of bone volume/total volume (BV/TV) (Fig. 5B), percentages of osteoid surface (OS/BS) (Fig. 5C) and bone surface covered with osteoblasts (Ob.S/BS) (Fig. 5D); we also found higher percentages of bone surface eroded by osteoclasts (ES/BS) (Fig. 5E) and bone surface covered with osteoclasts (Oc.S/BS) (Fig. 5F). In agreement with these findings, the bone formation rate was decreased in complete remission myeloma mice with *E. coli* infection (Fig. 5G, H). Thus, *E. coli* contributes to myeloma patients bone disease in complete remission.

**Fig. 6:**
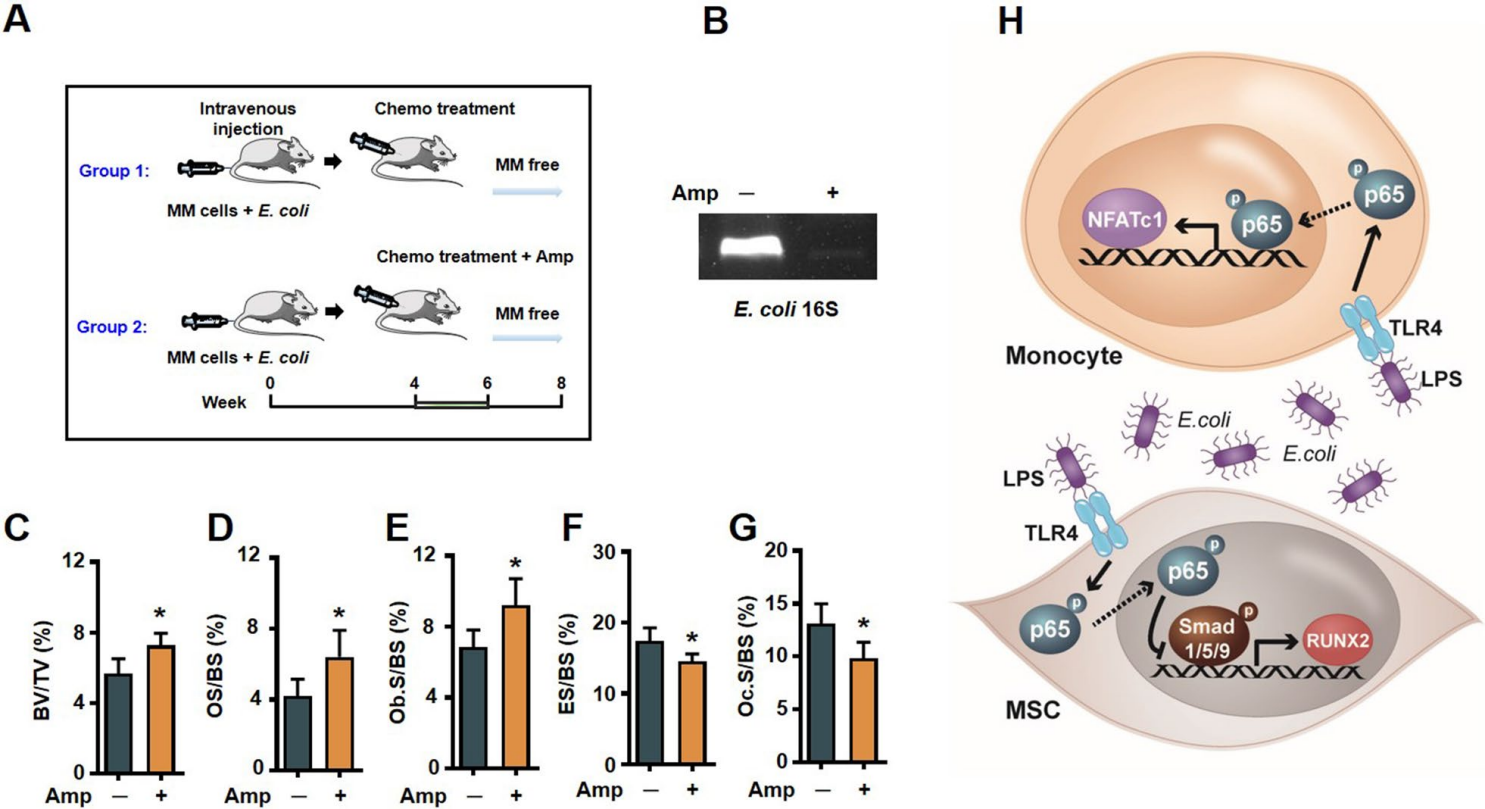
Elimination of *E. coli* heals bone resorption in a mouse model of myeloma in complete remission. **A** C57BL/KaLwRij mice were intravenous injected with 5TGM1 (5 × 10^5^ cells/mouse) and *E. coli* cells (1 × 10^4^ cells/mouse). After 4 weeks, 1 mg/kg bortezomib and 2 mg/kg melphalan with or without Amp (100 mg/kg) were injected intraperitoneally into the mice (3 times a week) for 2 weeks. **B** The expression of *E. coli 16S* rRNA in mice bone marrow aspirates. Histomorphometric analysis of BV/TV (**C**), OS/BS (**D**), Ob.S/BS (**E**), ES/BS (**F**) and Oc.S/BS (**G**). Data are means ± SD (*n* = 5 mice/group, three replicate studies). *P < 0.05. *P* values were determined using Student’s *t* test. **H** Depiction of signaling pathways involved in the *E. coli*-mediated suppression of osteoblastogenesis and activation of osteoclastogenesis

Toward a therapeutic, we asked whether elimination of *E. coli* in bone marrow helps healing of resorbed bone of myeloma in complete remission. We intravenous injected myeloma cells 5TGM1 with *E. coli*, 4 weeks later, treated the mice with chemotherapy drugs and with or without ampicillin (Fig. 6A). We observed *16S* rRNA gene expression of *E. coli* in bone marrow aspirates of mice without ampicillin treated group (Fig. 6B), which suggested that the ampicillin -treated mice were almost or totally *E. coli* free. Bone histomorphometric analysis demonstrated a higher BV/TV (Fig. 6C), OS/BS (Fig. 6D) and Ob.S/BS (Fig. 6E); lower ES/BS (Fig. 6F) and Oc.S/BS (Fig. 6G) in ampicillin-treated mice. These findings indicated that counteracting *E. coli* may be effective for prevention or treatment of osteolytic bone lesions persistence of myeloma patients in complete remission.

## Discussion

Myeloma cells disrupt the delicate balance between bone formation and resorption, leading to osteolytic bone lesions [2]. It is well established that myeloma cells enhance osteoclast differentiation and inhibit osteoblast differentiation via secreting osteolytic cytokines, but a confusing problem that has plagued the treatment osteolytic bone disease of patient with myeloma is why myelomatous bone lesions do not heal, even following successful treatment. Little attention has been given to the molecular mechanism of this problem. Previous studies described that myeloma cells induced aberrant DNA methylation in MSCs, which resulted in the dysregulation of osteogenesis [21]. Our previous study demonstrated that myeloma cells can reprogram bone marrow adipocytes via altered promoter histone methylation of the peroxisome proliferator-activated receptor γ (*PPARγ*), which reduced expression of PPARγ and a modified adipokine secretion profile, causing enhanced osteoclastogenesis and suppression of osteoblastogenesis in complete remission patient [20]. Here, through a combination of in vitro, in vivo, and patient studies, we clearly described a role for bone marrow bacteria in this process. We demonstrated that *E. coli* LPS stimulates bone resorption and inhibits bone formation via NF-κB p65 signaling. Further in vivo assay showed that antibiotics treatment is probably a therapeutic strategy for myeloma bone disease. Together, bacterial infection may be responsible, at least in part, for maintaining bone destruction in myeloma.

Previous researches have significantly broadened our understanding of how bacteria regulate bone remodeling. For instance, gut microbes play an important role in the development of bone-related diseases such as osteoporosis [22], and intestinal microorganisms affect bone metabolism by regulating growth factors [22]. Besides, LPS has been showed to inhibit osteoblast differentiation through Myd88-dependent signaling and induce the apoptosis and inhibit osteoblast differentiation through JNK pathway in MC3T3-E1 cells. Additionally, LPS induced the differentiation of human monocyte to osteoclast through tumor necrosis factor (TNF) α-dependent pathway [23]. In our work, we showed that phosphorylated NF-κB p65 inhibits RUNX2 expression by reducing phosphorylated smad1/5/9 binding ability with *RUNX2* promoter in osteoblast progenitors, and enhances NFATc1 expression by increasing phosphorylated NF-κB p65 binding ability with *NFATc1* promoter in osteoclast progenitors. But there are also limitations. Bone remodeling is a complex event in which multiple factors, regulating both formation and resorption, collaborate to maintain bone stability. Except LPS and osteoblast or osteoclast interactions, whether LPS can interact with other cells in bone marrow microenvironment and regulate bone lesions still need further investigation.

Collectively, our results elucidate a new mechanism for myelomatous bone lesions do not heal following successful treatment. These data demonstrate that bone marrow *E. coli* LPS inhibits osteoblastogenesis and enhances osteoclastogenesis through NF-κB p65 signaling (Fig. 6H). Eliminating *E. coli* infection in myeloma mouse model help heal bone resorption of myeloma in remission. These observations are important because they not only provide insight into a thornier issue but also offer a potential therapeutic strategy.

## Materials and methods

### Cell lines

*Escherichia coli* was purchased from the American Type Culture Collection (ATCC, Cat# 25922) and cultured in the Luria Bertani (LB) medium. The murine myeloma cell line 5TGM1 was provided by Dr. Zhiqiang Liu’s lab of Tianjin Medical University. The 5TGM1 cells were cultured in Iscove’s Modified Eagle’s Medium (IMDM) (Thermo Fisher Scientific, Cat# 31980030) with 10% fetal bone serum. Patient bone marrow biopsy samples were collected from the Qingdao Municipal Hospital of Qingdao University. Bone lesions numbers were characterized by radiologists at Qingdao Municipal Hospital. All experiments were performed with mycoplasma-free cells.

### Antibodies and reagents

All chemicals were purchased from Sigma-Aldrich, except where specified. BoneTRAP (TRACP 5b human) ELISA kit was purchased from immunodiagnostic systems (IDS) (Cat# SB-TR201R).

### In vitro osteoblast and osteoclast differentiation and function assays

MSCs were isolated from the bone marrow of healthy donors and cultured in osteoblast differentiation medium to obtain mature osteoblasts [20]. The bone formation activity of osteoblasts was demonstrated using Alizarin red S staining (Sigma-Aldrich, Cat# A5533). Human monocytes were isolated from peripheral blood mononuclear cells of health donors and cultured in M-CSF (25 ng/ml) (R&D Systems, Cat# 216-MC) for 7 days to obtain the precursors of osteoclasts. The precursors derived from human monocytes were cultured in M-CSF (25 ng/ml) and RANKL (10 ng/ml) (R&D Systems, Cat# 6449-TEC), cocultured with or without *E. coli* or LPS for 7 days to induce mature osteoclast formation. Leukocyte acid phosphatase kit (Sigma-Aldrich, Cat# 387A) for TRAP staining was used to detect mature osteoclasts.

### Western blot analysis

Cells were collected and lysed with lysis buffer (Cell Signaling Technology, Cat# 9803S). Cell lysates were subjected to SDS-PAGE assay, transferred to a Polyvinylidene fluoride (PVDF) membrane (Millipore, Cat# IPVH00010), and immunoblotted with antibodies against GAPDH (Cat# 2118), RUNX2 (Cat# 12556), p65 (Cat# 8242), phosphorylated p65 (Cat# 3033), phosphorylated smad1/5/9 (Cat# 13820) and NFATc1 (Cat# 8032) (Cell Signaling Technology).

### Quantitative real-time PCR of mRNAs

Total RNA was isolated using a Total-RNA isolation kit (Thermo Fisher Scientific, Cat# 12183025). An aliquot of 0.5 μg of total RNA was subjected to reverse transcription (RT) with a SuperScript II RT-PCR kit (Invitrogen, Cat# 18064014). Quantitative PCR was performed using SYBR Green Master Mix (Life Technologies, Cat# A25780) with the 7500 Real-Time PCR System (Life Technologies). The primers used are listed in Additional file 1: Table S1.

### ChIP assay

Cells were fixed in 4% formaldehyde and sonicated to prepare chromatin fragments using SimpleChIP Enzymatic Chromatin IP Kit (Cell Signaling Technology, Cat# 9002). Chromatin samples were immunoprecipitated with antibodies against phosphorylated p65 and phosphorylated smad1/5/9 and control IgG (Cell Signaling Technology, Cat# 2729S) at 4 °C for 4 h. Immunoprecipitates and total chromatin inputs were reverse cross-linked. DNA was isolated and analyzed using quantitative real-time PCR. The primer sequences used are listed in Additional file 1: Table S2. Relative fold enrichment was calculated by determining the immunoprecipitation efficiency (ratio of the amount of immunoprecipitated DNA to that of the input sample).

### Detection of *16S* rRNA genes

Genomic DNA (gDNA) was extracted from fresh bone marrow aspirates with the QIAamp DNA Mini Kit (QIAGEN, Cat# 51304). gDNA from each specimen was subjected to qPCR to determine the amounts of bacteria by detecting the 16S genes. qPCR was performed using SYBR Green Master Mix (Life Technologies, Cat# A25780) with the 7500 Real-Time PCR System (Life Technologies). The RNase P copy number assay was utilized as a basis to compare bacterial DNA content to host (human) DNA content by performing a logarithmic ratio of the cycle thresholds obtained for bacterial and host DNA. The primers used in RT-PCR and qPCR are in Additional file 1: Table S3.

### Hematoxylin and eosin (HE) staining and immunohistochemistry

Formalin-fixed, paraffin-embedded sections of bone marrow biopsy samples obtained from patients with myeloma were deparaffinized and stained. HE staining was performed according to standard protocols [24]. Slides were stained with anti-LPS (Cat# LS-C75640) and LTA (Cat# LS-C202488) (LifeSpan BioSciences) antibody using an EnVision System (DAKO, Cat# K5361) following the protocols and nuclei were counterstained with hematoxylin.

### In vivo mouse experiments, radiography and bone histomorphometry

C57BL/KaLwRij mice purchased from Charles River Labs, Beijing, China, were maintained in Xiamen University Animal Care-accredited facilities. 5TGM1 cells (5 × 10^5^ cells per mouse) were intravenous injected into 8-week-old C57BL/KaLwRij mice with or without *E. coli* (1 × 10^4^ cells per mouse). After 4 weeks, 1 mg/kg bortezomib (Cayman Chemicals, Cat# 10008822) and 2 mg/kg melphalan (Cayman Chemicals, Cat# 16665) were injected intraperitoneally into the mice three times a week for 2 weeks. Serum samples were collected from the mice weekly and tested for myeloma-secreted M proteins using ELISA analysis to monitor the tumor burden (Thermo Fisher Scientific, Cat # 88-50430-88). To examine the lytic bone lesions, radiographs were scanned with a Bruker In-Vivo Xtreme imaging system. Bone tissues were fixed in 4% paraformaldehyde and decalcified, and sections of them were stained with toluidine blue or TRAP following standard protocols. Both analyses were done using the BIOQUANT OSTEO software program (BIOQUANT Image Analysis Corporation).

### Statistical analysis

Statistical significance was analyzed using the Graphpad 9.0 program with Student *t*-tests for comparison of two groups, and one-way ANOVA with Tukey’s test for comparison of more than two groups. *P* values less than 0.05 were considered statistically significant. All results were reproduced in at least three independent experiments.

## Supplementary Information

**Additional file 1: Table S1.** Primers used in real-time quantitative PCR analysis. **Table S2.** Primers for ChIP-PCR. **Table S3.** *16S* rRNA primers.

## Data Availability

The data and materials that support the findings of this study are available from the corresponding author upon reasonable request.
