# Supplementary material for: Bacterial infections exacerbate myeloma bone disease
Source: J Transl Med. 2022 Jan 6;20:16. doi: 10.1186/s12967-021-03187-5 (PMC8734283; doi:10.1186/s12967-021-03187-5)
Supplement: Supplementary file 1 — Additional file 1: Table S1. Primers used in real-time quantitative PCR analysis. Table S2. Primers for ChIP-PCR. Table S3. 16S rRNA primers. [file 12967_2021_3187_MOESM1_ESM.docx]

**Additional file 1**

**Table S1. Primers used in real-time quantitative PCR analysis**

| **Gene** | **Forward** | **Reverse** |
| --- | --- | --- |
| *GAPDH* | CTGGGCTACACTGAGCACC | AAGTGGTCGTTGAGGGCAATG |
| *TRAP* | AGATCCTGGGTGCAGACTTC | AAGGGAGCGGTCAGAGAATA |
| *CALCR* | GGGAATCCAGTTTGTCGTCT | ACAAAGAAGCCCTGGAAATG |
| *CTSK* | CCATATGTGGGACAGGAAGA | CCTCTTCAGGGCTTTCTCAT |
| *BGLAP* | ACTGTGACGAGTTGGCTGAC | AAGAGGAAAGAAGGGTGCCT |
| *ALP* | TCCCAGTTGAGGAGGAGAAC | CCCAGGAAGATGATGAGGTT |
| *COL1A1* | TGTTCAGCTTTGTGGACCTC | GGTGATTGGTGGGATGTCTT |
| *RUNX2* | TCAACGATCTGAGATTTGTGGG | GGGGAGGATTTGTGAAGACGG |
| *NFATc1* | CACCGCATCACAGGGAAGAC | GCACAGTCAATGACGGCTC |

**Table S2. Primers for ChIP-PCR.**

| **Primers for binding site of:** | **Forward** | **Reverse** |
| --- | --- | --- |
| p-smad1/5/9 on *RUNX2* promoter | GAACCCACACCGCTTCACCA | TTTGATGAGGCCGACTGCCG |
| p-p65 on *NFATc1* promoter | CCTTAGGTACCTGGTGCCGC | CCGCCGGCTTCCGAGTTTTA |

**Table S3. *16S* rRNA primers**

| **Gene** | **Forward** | **Reverse** |
| --- | --- | --- |
| Universal *16S* | CCTACGGGNGGCWGCAG | GACTACHVGGGTATCTAATCC |
| *S. pneumoniae 16S* | ACGCAACTGACGAGTGTGAC | GATCGCGACACCGAACTAAT |
| *E. coli 16S* | CTCCTACGGGAGGCAGCAG | GWATTACCGCGGCKGCTG |

N: A/T/G/C W: A/T H: A/C/T V: A/G/C

W: A/T K: G/T
